# Evaluation of anti-citrullinated type II collagen and anti-citrullinated vimentin antibodies in patients with juvenile idiopathic arthritis

**DOI:** 10.1186/1546-0096-11-31

**Published:** 2013-08-29

**Authors:** Brooke E Gilliam, Anil K Chauhan, Terry L Moore

**Affiliations:** 1 Division of Adult and Pediatric Rheumatology, Saint Louis University School of Medicine, 1402 South Grand Blvd., Room 211A Doisy Hall, Saint Louis, Missouri 63104, USA; 2 Division of Adult and Pediatric Rheumatology, Internal Medicine, Pediatrics, and Molecular Microbiology and Immunology, Saint Louis University School of Medicine, Saint Louis, Missouri, USA

**Keywords:** Juvenile idiopathic arthritis, Anti-cyclic citrullinated peptide antibodies, Type II collagen, Vimentin

## Abstract

**Background:**

To determine the prevalence and significance of anti-citrullinated vimentin and anti-citrullinated type II collagen antibodies and elucidate their role in the disease process of juvenile idiopathic arthritis (JIA).

**Methods:**

Sera were obtained from 95 patients with various subtypes of JIA, 19 systemic lupus erythematosus (SLE) patients, and 10 healthy children. Antibodies were measured in the sera against citrullinated and native type II collagen and vimentin (vim1-16 and vim 59-74) by enzyme-linked immunosorbent assay. Samples were compared to anti-cyclic citrullinated peptide (anti-CCP) antibody and rheumatoid factor (RF) isotypes, and our previously measured anti-citrullinated fibrinogen and α-enolase antibodies on the same patient population, in addition to erythrocyte sedimentation rate and C-reactive protein. The relationship between the anti-citrullinated antibody profile and disease activity and joint damage were also investigated.

**Results:**

Twenty-three JIA patients (24%) demonstrated reactivity to anti-citrullinated type II collagen. Ten JIA patients (10.5%) demonstrated reactivity to anti-citrullinated vimentin 1–16 antibodies and 7 (7.4%) to anti-citrullinated vimentin 59–74 antibodies. One IgM RF-positive polyarticular patient was positive for all 5 of the citrullinated autoantibodies tested. Thirty-seven different subsets of patients were identified based on their anti-citrullinated autoantibody and RF isotype profile. No significant associations were noted with anti-citrullinated type II collagen and anti-citrullinated vimentin antibodies with joint damage or disease activity. Anti-citrullinated vimentin 59–74 antibodies demonstrated the highest overall specificity at 89.7%, with anti-citrullinated vimentin 1–16 and anti-citrullinated type II collagen antibodies at 86.2%.

**Conclusion:**

This study demonstrates that antibodies to multiple citrullinated epitopes are present in the sera of patients with various subtypes of JIA. It also demonstrates the frequent occurrence of anti-citrullinated type II collagen and anti-citrullinated fibrinogen antibodies. The presence of autoantibodies to citrullinated antigens in JIA patients is highly diverse.

## Background

Rheumatoid arthritis (RA) and certain subtypes of juvenile idiopathic arthritis (JIA) are manifested by the formation of autoantibodies. IgM rheumatoid factor (RF), determined by the latex fixation test (LFT), is the most well-characterized autoantibody and is included in the America College of Rheumatology/European League Against Rheumatism classification criteria for RA and the International League of Associations for Rheumatology (ILAR) criteria for the IgM RF-positive polyarticular JIA subtype [1], [2]. Anti-cyclic citrullinated peptide (anti-CCP) antibodies have been established as an important diagnostic tool in RA, especially in patients demonstrating a more severe, erosive disease course [3]. We and several other groups have shown that anti-CCP antibodies are present in JIA patients. They are associated with aggressive disease and manifested by various anti-CCP antibody isotypes. The IgM RF-positive polyarthritis subtype most closely resembles adult RA [4-10].

While the role of anti-CCP antibodies in RA and JIA has become better understood, the identity of the target proteins of the citrulline modification remains undetermined. Type II collagen is the most abundant protein in articular joints [11]. Type II collagen, when injected into genetically susceptible animals, induces collagen-induced arthritis (CIA) and is one of the common animal models for RA [12]. Anti-Sa antibodies, which react to citrullinated vimentin are highly specific for RA [13]. Few studies have evaluated the role of anti-citrullinated vimentin antibodies in JIA [14], [15]. There are no published studies evaluating the significance of anti-citrullinated type II collagen antibodies in JIA.

The aim of this study was to investigate the presence of anti-citrullinated antibodies reactive to various modified peptide epitopes, including anti-citrullinated type II collagen and two linear peptide epitopes derived from vimentin. Combined with our previous studies on anti-citrullinated fibrinogen and α-enolase antibodies with the same JIA population [9], we attempted to determine the prevalence and significance of previously identified target proteins for citrullination and to further elucidate their role in the JIA disease pathogenesis.

## Methods

### Patient samples

A previously described and studied patient and control population was used for the current study [9]. Sera were collected from 95 JIA patients (77 female/18 male) from the Saint Louis University Pediatric Rheumatology outpatient clinics at the Saint Louis University Medical Center and Cardinal Glennon Children’s Medical Center, following informed consent. JIA patient samples included 16 patients with IgM RF-positive polyarthritis, 36 with IgM RF-negative polyarthritis, 24 with oligoarthritis, 13 with systemic-onset arthritis, 3 with psoriatic arthritis, and 3 with enthesitis-related arthritis. All JIA patients in this study fulfilled ILAR criteria [1], [2]. JIA patient demographics are listed in Table 1. Sera from 19 childhood-onset systemic lupus erythematosus (SLE) patients (17 female/2 male) were collected from the outpatient clinics, following informed consent. The mean age of the SLE patients was 15.7±3.1 years and the mean disease duration was 2.7±3.2 years. Sera were also collected from 10 healthy children (9 female/1 male) at the well-child clinic at Cardinal Glennon Children’s Medical Center, following informed consent. The mean age for the healthy children was 14.0±5.9 years. The study was approved by the Institutional Review Board of the Saint Louis University Medical Center.

**Table 1:** Demographic and laboratory features, given by median (interquartile range), of patients stratified by JIA subtype

	**JIA**	**IgM RF+ polyarthritis**	**IgM RF- polyarthritis**	**Oligoarthritis**	**Systemic arthritis**	**Psoriatic arthritis**	**Enthesitis-related arthritis**
	**n=95**	**n=16**	**n=36**	**n=24**	**n=13**	**n=3**	**n=3**
Sex, no. females/males	77/18	14/2	30/6	22/2	8/5	3/0	0/3
Age, median (IQ range)	12 (5–16)	14.5 (9.8-16)	12 (5.3-15.5)	10 (4.3-12)	10.5 (4.8-14.3)	17 (15–17)*	16 (5–17)*
Disease duration, median (IQ range)	2 (0.5-6.5)	0.5 (0.4-5.1)	2.3 (0.6-6)	2 (0.6-8.1)	3.8 (1–9.5)	4 (0–8)*	2.5 (1–7)*
Tender/swollen joint count, median (IQ range)	8 (2–10)	10 (8–13)	8 (6–12)	1 (1–2)	9 (1–22)	2 (1–8)*	2 (1–2)*
No. patients with joint damage (%)	20 (20.8)	5 (31.3)	10 (27.8)	1 (4.2)	4 (30.8)	0 (0.0)	0 (0.0)
CRP, median (IQ range) mg/dl	0.7 (0.3-1.9)	2.6 (0.8-13.3)	0.80 (0.3-4.8)	0.8 (0.3-5.5)	4.9 (2.2-16.3)	0.7 (0.33-2.1)*	0.4 (0.3-2.7)*
(% positive)	(36.8)	(50.0)	(33.3)	(25.0)	(29.2)	(33.3)	(33.3)
ESR, median (IQ range) mm/hr	16 (7–37)	20 (8–31)	14 (7–32)	15 (7–31)	30 (7–50)	6 (6–32)*	7 (2–7)*
(% positive)	(50.5)	(68.8)	(44.4)	(45.8)	(69.2)	(33.3)	(0.0)

*CRP*: C-reactive protein; *ESR*: Erythrocyte sedimentation rate.

Cut-points for a positive value: CRP (≥0.8 mg/dl), ESR (≥15 mm/hr). *range is given due to small sample size.

At the time of sample collection, 38 JIA patients were taking non-steroidal anti-inflammatory drugs [33 on naproxen, 2 on nabumetone, one on diclofenac, one on celecoxib, and one on tolmetin], 35 were taking disease modifying anti-rheumatic drugs [31 on methotrexate, 2 on leflunomide, and 2 on sulfasalazine], 15 were treated with biologics [12 with etanercept, one with infliximab, one with abatacept, and one with anakinra], 9 JIA patients were taking hydroxychloroquine, 6 patients were taking prednisone, and one was taking prednisolone. Eighteen JIA patients were not taking any medication at the time of sample collection, as this was either their initial visit to the rheumatology clinic or they had been lost to follow up and later returned to the clinic.

### Laboratory and clinical evaluation

Erythrocyte sedimentation rate (ESR) was determined by modified Westergren technique and considered elevated at ≥15 mm/hr. C-reactive protein (CRP) was determined by electroimmunoassay and a value of ≥0.8 mg/dl was considered elevated. Initial determination of IgM RF positivity was performed using the latex fixation test (LFT), which is how seropositive JIA patients were classified. The QUANTA-Lite RF ELISA (INOVA Diagnostics, Inc., San Diego, CA) was used for detection of IgA and IgM RF following manufacturer’s instructions. The cut-off value for positive IgA or IgM RF was 6 U. A third generation anti-CCP antibody test, the QUANTA-Lite CCP3 ELISA (INOVA Diagnostics, Inc.) was used for detection of IgG anti-CCP antibodies according to manufacturer’s instructions. The cut-off value for a positive result was 20 U. IgA and IgM anti-CCP antibodies were measured as previously described [10]. Cut-off values for a positive result were calculated at optical density (OD) = 0.16 and OD = 0.43, respectively.

Sixty-six JIA patients had active disease at the time of sample collection and 29 were in disease remission. Clinical data regarding signs of active disease (including joint pain and swelling, limitations of range of motion, fever, rash, visceral involvement, and inflammatory markers) were collected from patient records of the Pediatric Rheumatology clinics. Joint damage was noted in 20/95 JIA patients. Radiological data was evaluated for signs of joint damage (defined as joint space narrowing and/or erosions) by musculoskeletal radiologists and reviewed by pediatric rheumatologists. Both clinical and laboratory data were collected from the same time period as sera were collected.

### In vitro deimination of type II collagen

Type 2 rabbit skeletal muscle peptidyl arginine deiminase (PAD) (Sigma, Saint Louis, MO) was used for enzymatic treatment of human type II collagen (Chondrex, Redmond, VA) following a previously described protocol [16]. Human type II collagen was incubated at a concentration of 20 μg/ml with 2 U/ml PAD at 37°C for 18 hours in a buffer of 20 mM Hepes (pH 8.8), 0.3 M NaCl, 1 mM EDTA, 1 mM dithiothreitol (DTT), and 10 mM CaCl_2_. EDTA was added to stop the reaction (10 mM final concentration).

### Citrullinated and native autoantibody ELISAs

The ELISA for measurement of anti-citrullinated type II collagen antibodies was performed as previously described, with modifications [16]. Briefly, 96-well microtitre plates (Nunc, Roskilde, Denmark) were coated with native or citrullinated type II collagen (10 μg/ml) and blocked with 2% bovine serum albumin (BSA) in phosphate buffered saline (PBS) for one hour at 4°C, followed by three washes with PBS/0.05% Tween. Sera were diluted 1:50 in radioimmunoassay (RIA) buffer (1% BSA, 350 mM NaCl, 10 mM Tris–HCl pH 7.6, 1% vol/vol Triton X-100, 0.5% wt/vol Na-deoxycholate, 0.1% SDS) added to the wells in duplicate, and incubated for two hours at room temperature (RT) with gentle agitation. After 3 washes with PBS/0.05% Tween, goat anti-human IgG horseradish peroxidase (HRP) (Antibodies Incorporated, Davis, CA) diluted in RIA buffer was added to the wells at a concentration of 1:10,000 and incubated for one hour at RT. After a final wash step, bound antibodies were detected with tetramethylbenzidine (TMB), an HRP substrate (BioFX, Owing Mills, Maryland), and the reaction was stopped with the addition of 0.25 M H_2_SO_4_. The absorbance was measured at 450 nm (Tecan Group Ltd., Männedorf, Switzerland). Patient results from duplicate wells were averaged and the OD from blank wells containing PBS/0.05% Tween were subtracted from the average. Serum was considered positive if the titer reached two standard deviations (SD) above the mean for healthy controls. Positive cut-off points were OD = 3.03 for anti-citrullinated type II collagen antibodies and OD = 3.3 for native type II collagen.

Antibodies against the citrullinated and native form of two linear peptides derived from vimentin ((Vim) amino acids (aa) 1–16 STCitS VSSS SYCitCit MFGG and Vim aa 59–74 VYAT CitSSA VCitLCit SSVP) were determined by ELISA, as previously described [17]. For native vimentin, arginine replaced citrulline for each peptide sequence. The vimentin epitopes used have been frequently recognized by serum from anti-CCP antibody positive RA patients with longstanding disease [17]. Briefly, 96-well microtitre plates were coated with native or citrullinated vimentin peptide (10 μg/ml) in PBS/0.1% BSA and incubated overnight at 4°C. Sera were diluted 1:100 in PBS/1% BSA/0.05% Tween, added in duplicate, and incubated for 60 minutes at 37°C with gentle agitation. After three washes with PBS/0.05% Tween, rabbit anti-human IgG HRP (Antibodies Incorporated) diluted in RIA buffer was added to the wells at a concentration of 1:10,000 and incubated for one hour. After a final wash step, bound antibodies were detected with TMB, and the reaction was stopped with the addition of 0.25M H_2_SO_4_. The absorbance was measured at 450nm. Patient results from the duplicate wells were averaged and the OD from a blank well containing PBS/0.05% Tween was subtracted from the average. Serum was considered positive if the titer reached two SD above the mean for healthy controls. Positive cut-off points were OD = 0.79 for anti-citrullinated vimentin aa 1–16 antibodies and OD = 1.3 for native vimentin aa 1–16. Positive cut-off points were OD = 0.81 for anti-citrullinated vimentin aa 59–74 antibodies and OD = 0.83 for native vimentin aa 59–74.

### Statistical analysis

Patient groups were compared using Student’s t test and χ^2^ test for proportions. For tables with cells with small frequencies, Fisher’s exact test was used. Correlations were analyzed by Spearman’s rho correlation coefficient. Correlations were described as either strong (>0.7), moderate (0.7-0.5), fair (0.49-0.3), or poor (<0.3). The sensitivity, specificity, and positive predictive value (PPV) of citrullinated type II collagen, and citrullinated vimentin aa 1–16 and aa 59–74 were calculated in the JIA population. The sensitivity expresses the percentage of JIA patients positive for the test and specificity expresses the frequency of negative tests in the absence of JIA or JIA subtypes. PPV describes the group of patients with a positive test result who are correctly diagnosed. Statistical analyses were carried out using SPSS version 19.0 (Chicago, IL). A p-value<0.05 was considered statistically significant.

## Results

### JIA serum reactivity to native and citrullinated type II collagen and vimentin

Twenty-three JIA patients (24.2%) showed reactivity to citrullinated type II collagen (Table 2). IgM RF-negative polyarthritis and oligoarthritis patients demonstrated the highest level of reactivity against citrullinated type II collagen (Table 2). Twelve of the 23 (52.2%) JIA patients positive for anti-citrullinated type II collagen antibodies were also positive for IgM RF, which was measured by ELISA, but only in two that were positive by LFT. Seventeen (17.9%) JIA patients reacted with native type II collagen, including one with psoriatic arthritis, 2 with IgM RF-positive polyarthritis, 7 with IgM RF-negative polyarthritis, and 7 with oligoarthritis. Thirteen of 23 (56.5%) for JIA patients positive for citrullinated type II collagen antibodies were also positive for native type II collagen antibodies. Three SLE patients (15.8%) and one healthy child (10.0%) were positive for citrullinated type II collagen, while 1 SLE patient (5.3%) and none of the healthy children reacted with native type II collagen.

**Table 2:** Anti-citrullinated type II collagen and vimentin antibody concentration and positivity in JIA subtypes (n=95)

	**Poly RF+**	**Poly RF-**	**Oligo**	**Systemic**	**Enthesitis**	**Psoriatic**
	**n=16**	**n=36**	**n=24**	**n=13**	**n=3**	**n=3**
	**Mean±SD**	**Mean±SD**	**Mean±SD**	**Mean±SD**	**Mean±SD**	**Mean±SD**
**Citrullinated Type II Collagen (OD)**	1.7±0.77	2.0±1.0	2.4±0.76	1.9±1.0	3.0±0.32	1.5±1.7
**Positivity n(%)**	2 (12.5%)	9 (25%)	7 (29.2%)	2 (15.4%)	2 (66.7%)	1 (33.3%)
**Native Type II Collagen (OD)**	1.4±0.82	1.8±1.1	2.4±0.97	1.5±0.78	2.4±0.60	1.4±1.7
**Postivity n(%)**	2 (12.5%)	7 (19.4%)	7 (29.2%)	0 (0%)	0 (0%)	1 (33.3%)
**Citrullinated Vimentin 1–16 (OD)**	0.63±0.79	0.47±0.17	0.45±0.30	0.49±0.25	0.38±0.12	0.34±0.08
**Positivity n(%)**	1 (6.25%)	3 (8.3%)	3 (12.5%)	3 (23.1%)	0 (0%)	0 (0%)
**Native Vimentin 1–16 (OD)**	0.67±0.80	0.50±0.17	0.47±0.29	0.55±0.26	0.42±0.12	0.35±0.07
**Positivity n(%)**	1 (6.25%)	0 (0%)	1 (4.2%)	0 (0%)	0 (0%)	0 (0%)
**Citrullinated Vimentin 59–74 (OD)**	0.74±0.80	0.55±0.22	0.51±0.24	0.46±0.2	0.45±0.12	0.36±0.1
**Positivity n(%)**	2 (12.5%)	2 (5.6%)	2 (8.3%)	1 (7.7%)	0 (0%)	0 (0%)
**Native Vimentin 59–74 (OD)**	0.69±0.82	0.44±0.20	0.38±0.22	0.42±0.18	0.26±0.11	0.29±0.05
**Positivity n(%)**	1 (6.25%)	1 (2.8%)	1 (4.2%)	0 (0%)	0 (0%)	0 (0%)

Continuous variables are expressed as means ± SD. *N*: Number of patients; *OD*: Optical density; *RF*: Rheumatoid factor. Cut points for positive values: Anti-citrullinated type II collagen antibodies (OD≥3.03), Native type II collagen antibodies (OD≥3.3), anti-citrullinated vimentin 1–16 antibodies (OD≥0.79), native vimentin 1–16 antibodies (OD≥1.3), anti-citrullinated vimentin 59–74 antibodies (OD≥0.81), native vimentin 59–74 antibodies (OD≥0.83).

Ten JIA patients (10.5%) were considered positive for anti-citrullinated vimentin aa 1–16 antibodies and seven (7.4%) were positive for anti-citrullinated vimentin aa 59–74 antibodies (Table 2). Four of the 10 (40.0%) JIA patients’ positive for anti-citrullinated vimentin aa 1–16 antibodies and 6/7 positive for anti-citrullinated vimentin aa 59–74 antibodies (85.7%) were also positive for IgM RF. Positivity for anti-citrullinated vimentin antibodies were found in all JIA subtypes, except psoriatic and enthesitis-related JIA. Two (2.1%) JIA patients reacted with native vimentin aa 1–16, including one with IgM RF-positive polyarthritis, one with IgM RF-negative polyarthritis and one with oligoarthritis. Three (3.2%) JIA patients reacted with native vimentin aa 59–74, including one with IgM RF-positive polyarthritis and one with oligoarthritis. All JIA patients positive for native vimentin antibodies were also positive for citrullinated vimentin antibodies. Four (21.1%) SLE patients reacted with citrullinated vimentin aa 1–16, while two (10.5%) reacted with native vimentin aa 1–16. None of the healthy individuals reacted with citrullinated vimentin aa 1–16 and two (20.0%) were positive for native vimentin aa 1–16. Anti-citrullinated vimentin aa 59–74 antibodies were found in 3 (15.8%) SLE patients and none of the healthy individuals. Three SLE (15.8%) and three healthy children (30.0%) reacted with native vimentin aa 59–74.

One oligoarthritis patient was positive for anti-citrullinated type II collagen antibodies, and both anti-citrullinated vimentin aa 1–16 and aa 59–74. When all citrullinated autoantibodies were considered, including anti-citrullinated fibrinogen and α-enolase antibodies, measured in our previously published study [9], one IgM RF-positive polyarthritis patient reacted to all 5 of the anti-citrullinated autoantibodies tested.

### Commonality between anti-citrullinated antibody reactivities in JIA

**Figure 1:**
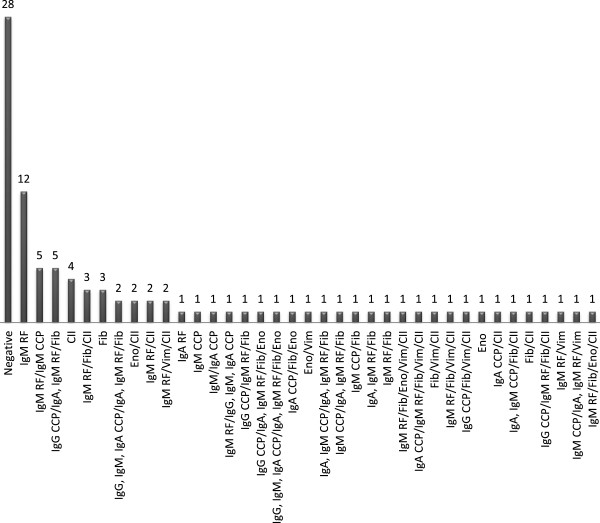
**Patterns of citrullinated antibody reactivity in JIA sera (n=95).** CII: anti-citrullinated type II collagen antibodies; CCP: cyclic citrullinated peptide; Eno: anti-citrullinated α-enolase antibodies; Fib: anti-citrullinated fibrinogen antibodies; RF: rheumatoid factor; Vim: anti-citrullinated vimentin (aa1-16 and aa59-74) antibodies.

Antibodies against various citrullinated proteins may be present in the sera at the same time. Thirty-seven different subsets of patients were identified based on their anti-citrullinated autoantibody and RF isotype profile, in addition to a group who were negative for all measured autoantibodies (Figure 1). IgM RF positivity alone was observed most frequently (n=12), followed by both IgM RF/IgM anti-CCP antibody positivity (n=5) and IgG anti-CCP antibodies/IgA and IgM RF/anti-citrullinated fibrinogen antibody positivity (n=5).

### Serological correlations

Anti-citrullinated type II collagen antibodies demonstrated a fair correlation with anti-citrullinated vimentin aa 1–16 and aa 59–74, anti-citrullinated α-enolase antibodies, and anti-citrullinated fibrinogen antibodies (Table 3). Anti-citrullinated vimentin aa 1–16 also correlated strongly with anti-citrullinated vimentin aa 59–74 (r=0.96, p<0.001).

**Table 3:** Correlations between the panel of citrullinated autoantibodies and anti-CCP antibody isotypes

	**Vimentin 1-16**	**Vimentin 59-74**	**α-Enolase**	**Fibrinogen**	**IgG CCP**	**IgA CCP**	**IgM CCP**
**Type II Collagen**	0.41**	0.47**	0.31**	0.45**	0.21*	0.22*	NS
**Vimentin 1-16**		0.96**	0.39**	0.42**	0.33**	0.26*	0.24*
**Vimentin 59-74**			0.35**	0.39**	0.22*	0.23*	0.22*
**α-Enolase**				0.30*	0.24*	0.29*	NS
**Fibrinogen**					0.47**	0.28*	NS
**IgG CCP**						0.34**	NS
**IgA CCP**							0.43**

*CCP*: Cyclic citrullinated peptide. P-value <0.05 indicated by *, p-value <0.001 indicated by **.

Anti-citrullinated vimentin aa 1–16 and aa 59–74 antibodies correlated significantly with IgM anti-CCP antibodies (r=0.24, p=0.01 and r=0.22, p=0.018, respectively) and IgA anti-CCP antibodies (r=0.26, p=0.006 and 0.23, p=0.014, respectively), though the correlation was poor. IgG anti-CCP antibodies correlated significantly with anti-citrullinated vimentin aa 1–16 (r=0.33, p<0.001), anti-citrullinated vimentin aa 59–74 (r=0.22, p=0.022), and citrullinated type II collagen (r=0.21, p=0.026). IgM RF demonstrated a significant but poor correlation with anti-citrullinated vimentin aa 1–16 (r=0.26, p=0.006) and anti-citrullinated vimentin aa 59–74 (r=0.23, p=0.014). Anti-citrullinated vimentin aa 1–16 also correlated significantly with IgA RF (r=0.22, p=0.017).

### Relationship between serological markers and disease course

**Figure 2:**
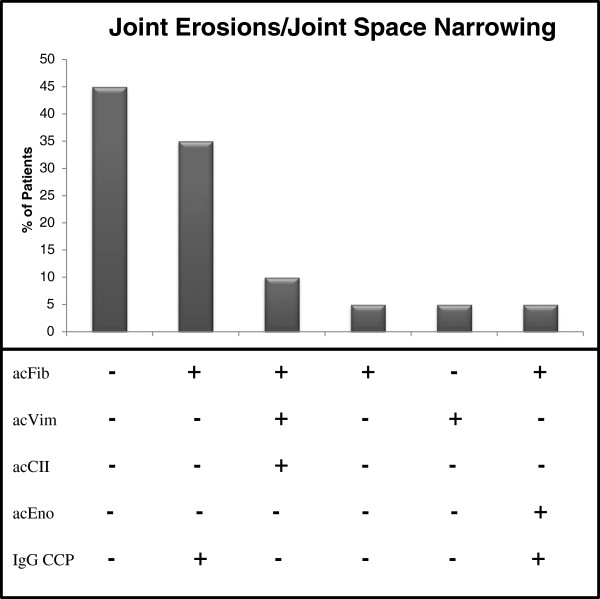
**Percentage of JIA patients with radiographic progression (n=20) based on their anti-citrullinated antibody profile.** acCII: anti-citrullinated type II collagen antibodies; acEno: anti-citrullinated α-enolase antibodies; acFib: anti-citrullinated fibrinogen antibodies; acVim: anti-citrullinated vimentin (aa1-16 and aa59-74) antibodies; CCP: cyclic citrullinated peptide.

JIA patients with joint damage were positive for various combinations of anti-citrullinated antibodies, with IgG anti-CCP and anti-citrullinated fibrinogen antibody positivity demonstrated in 7/20 (35%) patients with joint damage (Figure 2). Anti-citrullinated type II collagen antibodies and anti-citrullinated vimentin aa 1–16 and aa 59–74 antibodies were not significantly elevated in JIA patients with joint damage compared to those with no joint damage. When evaluating the relationship between the autoantibody profile and the number of joints damaged in JIA patients, a correlation was noted with IgA RF (r=0.38, p=0.001), IgM RF (r=0.43, p<0.001), anti-citrullinated fibrinogen antibodies (r=0.33, p=0.006), and IgA and IgG anti-CCP antibodies (r=0.26 and r=0.27, respectively, p<0.028). Levels of IgA (OD=0.17 vs. OD=0.04), IgG (46U vs. 5U), and IgM (OD=0.61 vs. OD=0.24) anti-CCP antibodies, IgA (9U vs. 1U) and IgM (32U vs. 6U) RF, and anti-citrullinated fibrinogen (OD=0.95 vs. OD=0.45) were significantly higher in patients with more 5 or more joints damaged compared to those with less than 5 joints damaged, respectively (p<0.03). No significant differences were noted for anti-citrullinated vimentin or type II collagen when evaluating the number of joints damaged in JIA patients. Nearly 50% of JIA patients with joint damage did not show any positivity for the antibodies tested (Figure 2).

It was noted that anti-citrullinated vimentin aa 1–16 antibodies demonstrated a faircorrelation with ESR (r=0.33, p=0.001). ESR levels were significantly elevated in JIA patients that were positive for anti-citrullinated vimentin aa 1–16 antibodies (46 mm/hr) compared to those who were negative (25 mm/hr; p=0.04).

### Sensitivity and Specificity of anti-citrullinated vimentin and type II collagen antibodies for JIA

The sensitivity, specificity, and PPV of anti-citrullinated vimentin aa 1–16 and aa 59–74 antibodies and anti-citrullinated type II collagen antibodies are shown in Table 4. Sensitivities, specificities, and PPVs were calculated for the overall JIA population and for each subtype, excluding enthesitis-related JIA and psoriatic JIA due to small samples size. Overall sensitivities for these parameters ranged from 7.8% to 25.8% and specificities from 86.2% to 89.7%. As with the sensitivities for JIA overall, sensitivities for anti-citrullinated type II collagen and vimentin antibodies in each JIA subtype remained low. Specificities were particularly high for anti-citrullinated vimentin aa 59–74 antibodies for all JIA subtypes included in this analysis (Table 4).

**Table 4:** Sensitivity, specificity, and PPV of anti-citrullinated antibodies for overall JIA population and JIA subtypes

	**Sensitivity (%)**	**Specificity (%)**	**PPV (%)**
	**With 95% CI**	**With 95% CI**	**With 95% CI**
**Anti-citrullinated type II collagen**			
**Antibodies (> 3.03OD)**			
Overall (n=95)	25.8 (17.4-36.4)	86.2 (67.4-95.5)	85.2 (65.4-95.1)
Poly RF-positive (n=16)	12.5 (2.2-39.6)	71.2 (59.3-80.9)	8.7 (1.5-29.5)
Poly RF-negative (n=36)	28.1 (14.4-47.0)	75.4 (61.9-85.5)	39.1 (20.5-61.2)
Oligoarthritis (n=24)	30.4 (14.1-53.0)	75.8 (63.4-85.1)	30.4 (14.1-53.0)
Systemic-Onset (n=13)	16.7 (2.9-49.1)	72.7 (61.2-82.1)	8.7 (1.5-29.5)
**Anti-citrullinated vimentin**			
**Antibodies 1–16 (> 0.79OD)**			
Overall (n=95)	10.9 (5.6-19.5)	86.2 (67.4-95.5)	71.4 (42.0-90.4)
Poly RF-positive (n=16)	6.3 (0.3-32.2)	88.2 (78.2-94.1)	10.0 (0.5-45.9)
Poly RF-negative (n=36)	9.1 (2.4-25.5)	88.1 (76.5-94.7)	30.0 (8.1-64.6)
Oligoarthritis (n=24)	12.5 (3.3-33.5)	89.7 (79.3-95.4)	30.0 (8.1-64.6)
Systemic-Onset (n=13)	23.1 (6.2-54.0)	91.1 (82.0-96.1)	30.0 (8.1-64.6)
**Anti-citrullinated vimentin**			
**Antibodies 59–74 (> 0.81OD)**			
Overall (n=95)	7.8 (3.5-15.9)	89.7 (71.5-97.3)	70.0 (35.4-91.9)
Poly RF-positive (n=16)	14.3 (2.5-43.8)	93.4 (84.7-97.6)	28.6 (5.1-69.7)
Poly RF-negative (n=36)	6.1 (1.1-21.6)	91.2 (80.0-96.7)	28.6 (5.1-69.7)
Oligoarthritis (n=24)	8.3 (1.5-28.5)	92.4 (82.5-97.2)	28.6 (5.1-69.7)
Systemic-Onset (n=13)	7.7 (0.4-37.9)	92.2 (83.2-96.8)	14.3 (0.8-58.0)

*CI*: Confidence interval; *OD*: Optical density; *PPV*: Positive predictive value; *RF*: Rheumatoid factor. Subtypes with small sample size were not included in the subtype analysis.

## Discussion

Several studies have attempted to identify the citrullination targets of proteins in RA and JIA [9], [18]. We previously evaluated sera from JIA patients for the presence of antibodies to citrullinated fibrinogen and α-enolase [9]. Both of these proteins are targets of the citrulline modification in RA [18-21]. In the present study, we built on this evaluation by further analyzing for the presence of anti-citrullinated type II collagen and vimentin antibodies.

The prevalence of anti-citrullinated vimentin antibodies in our JIA cohort was similar to the findings in two other studies, with positivity in 5.4% and 8.9% of their JIA population [14], [15]. Of note, the two studies evaluating anti-citrullinated vimentin antibodies in JIA used the commercially available anti-mutated citrullinated vimentin (MCV) ELISA (Orgentec, Mainz, Germany), producing similar results to the present study, using linear peptides derived from vimentin. Morbach et al. [15] reported sensitivity for anti-MCV antibodies for the diagnosis of IgM RF-positive polyarthritis to be 83.3% with specificity at 91.1%. The specificity in the present study (89.7%-86.2%) was comparable to that reported by Morbach et al. [15]; however, the sensitivity of their results was substantially higher. This observed difference in sensitivities may be due to different assays used for measurement of anti-citrullinated vimentin antibodies. However, Snir et al. [18] noted a high correlation between antibody responses to MCV and citrullinated vimentin peptides in both serum and synovial fluid.

The presence of anti-citrullinated vimentin antibodies did not correlate with radiological progression or disease activity in this JIA cohort; however, a fair correlation was made between anti-citrullinated vimentin aa 1–16 antibodies and ESR.Kuna et al. [14] reported no correlations between anti-MCV antibodies and ESR, tender/swollen joint count, or the Sharp score. However, Innala et al. [22] found that anti-MCV antibodies best identified patients with early RA who had persistent inflammatory activity, measured by Disease Activity Score (DAS)28, ESR, CRP and swollen joint count when compared to other antibodies against citrullinated peptide proteins. The same study noted no difference in predicting radiographic progression by measuring anti-MCV antibodies, second and third generation anti-CCP antibodies, IgA/IgG anti-CCP antibodies, or IgM RF [22], similar to findings by van der Linden et al [23].

The most commonly used mouse model for adult RA, CIA, uses immunization with type II collagen to induce arthritis [12]. However, only a small number of studies have analyzed the autoimmune response to native type II collagen in JIA and its potential to tolerize animals via oral administration of type II collagen [24-26]. Lindsley et al. [24] showed that antibodies to native type II collagen could be found in 33% of oligoarticular JIA patients and 14% of systemic-onset JIA patients, with Myers et al. [25] reporting that 72% of their JIA patient population had a significant inflammatory T cell response to type II collagen. These previously reported findings may explain the higher prevalence of native type II collagen antibodies (17.9%) in our JIA patient population. To date, there is no published data on the prevalence or significance of anti-citrullinated type II collagen antibodies in JIA, while this has been well-studied in adult RA [11], [12], [16]. The prevalence of autoantibodies to citrullinated type II collagen antibodies appears to be higher in adult RA patients compared to our JIA population, with one study detecting them in 78.5% of their RA patients [11], and another in 40.4% of RA patients [16]. It would be of interest to evaluate anti-citrullinated type II collagen antibody levels in the synovium of JIA patients, as it was shown in adult RA patients that citrullinated type II collagen was produced in the inflamed articular synovium [11]. Anti-citrullinated type II collagen antibodies were one of the most commonly observed citrullinated autoantibody in this JIA population, which also demonstrated a relatively high specificity for JIA (86.2%). Based on these findings, antibodies to both native and citrullinated type II collagen may play a role in JIA.

There were some limitations to the present study, including the small size of the healthy population used to generate cut-off values for anti-citrullinated type II collagen and vimentin antibodies. However, the prevalence of citrullinated autoantibodies in our cohort was similar to findings in previous studies [14], [15]. The enthesitis-related and psoriatic arthritis groups were rather small, with larger populations needed in future studies to confirm the significance of citrullinated type II collagen and vimentin autoantibodies in these subtypes. With the addition of more enthesitis-related arthritis patients in future studies, it will be interesting to determine if the high prevalence of anti-citrullinated type II collagen antibodies remains, or if this was a reflection of small sample size. Longitudinal studies with new or early-onset JIA will be necessary to further characterize the role these citrullinated autoantibodies play in aggressive disease, including joint damage.

## Conclusions

The present study showed that anti-citrullinated type II collagen and anti-citrullinated vimentin antibodies are present in JIA patients. The anti-citrullinated type II collagen and fibrinogen antibodies were the most frequently detected of the citrullinated autoantibodies in this JIA population. Overall, the presence of citrullinated autoantibodies in JIA patients is highly diverse, with several patterns of anti-citrullinated autoantibodies noted.

## Abbreviations

BSA: Bovine serum albumin; CCP: Cyclic citrullinated peptide; CIA: Collagen-induced arthritis; CRP: C-reactive protein; DAS: Disease activity score; DTT: Dithiothreitol; ELISA: Enzyme-linked immunosorbent assay; ESR: Erythrocyte sedimentation rate; HRP: Horseradish peroxidase; ILAR: International League of Associations for Rheumatology; JIA: Juvenile idiopathic arthritis; MCV: Mutated citrullinated vimentin; OD: Optical density; PAD: Peptidyl arginine deiminase; PBS: Phosphate buffered saline; PPV: Positive predictive value; RA: Rheumatoid arthritis; RIA: Radioimmunoassay buffer; RF: Rheumatoid factor; RT: Room temperature; SLE: Systemic lupus erythematosus; SPSS: Statistical package social sciences; TMB: Tetramethylbenzidine.

## Competing interests

The authors declare that they have no competing interests.

## Authors’ contributions

BEG conceived the study, participated in the design of the study, performed immunoassays, performed data analysis, and drafted the manuscript. AKC participated in the design of the study. TLM conceived the study, participated in the design of the study, and drafted the manuscript. All authors have read and approved the final manuscript.

## Authors’ information

**BEG**, M.S., is a Research Assistant at the Division of Adult and Pediatric Rheumatology, Saint Louis University School of Medicine. **AKC**, Ph.D., is a Visiting Professor at the Division of Adult and Pediatric Rheumatology, Saint Louis University School of Medicine. **TLM**, M.D., is the Director, Division of Adult and Pediatric Rheumatology and a Professor of Internal Medicine, Pediatrics, and Molecular Microbiology and Immunology at Saint Louis University School of Medicine.

## References

[B1] Aletha D Neogi T Silman AJ Funovits J Felson DT Bingham CO III Birnbaum NS Burmester GR Bykerk VP Cohen MD Combe B Costenbader KH Dougados M Emery P Ferraccioli G Hazes JM Hobbs K Huizinga TW Kavanaugh A Kay J Kvien TK Laing T Mease P Ménard HA Moreland LW Naden RL Pincus T Smolen JS Stanislawska-Biernat E Symmons D Tak PP Upchurch KS Vencovsky J Wolfe F Hawker G Rheumatoid arthritis classification criteria: an American college of rheumatology/European league against rheumatism collaborative initiative Ann Rheum Dis 2010 2010 69 1580 1588 10.1136/ard.2010.138461 20699241

[B2] Petty RE Southwood TR Manners P Baum J Glass DN Goldenberg J He X Maldonado-Cocco J Orozco-Alcala J Prieur AM Suarez-Almazor ME Woo P International League of Associations for Rheumatology: International League of Associations for Rheumatology classification of juvenile idiopathic arthritis: second revision, Edmonton, 2001 J Rheumatol 2004 31 390 392 14760812

[B3] Klareskog L Rönnelid J Lundberg K Padyukov L Alfredsson L Immunity to citrullinated proteins in rheumatoid arthritis Annu Rev Immunol 2008 26 651 675 10.1146/annurev.immunol.26.021607.090244 18173373

[B4] Omar A Abo-Elyoun I Hussein H Nabih M Atwa H Gad S Emad Y Anti-cyclic citrullinated peptide (anti-CCP) antibody in juvenile idiopathic arthritis (JIA): correlations with disease activity and severity of joint damage (a multicenter trial) Joint Bone Spine 2013 80 38 43 10.1016/j.jbspin.2012.03.008 22575064

[B5] Habib HM Mosaad YM Youssef HM Anti-cyclic citrullinated peptide antibodies in patients with juvenile idiopathic arthritis Immuno Invest 2008 37 849 857 10.1080/08820130802438057 18991100

[B6] Gilliam BE Chauhan AK Low JM Moore TL Measurement of biomarkers in juvenile idiopathic arthritis patients and their significant association with disease severity: a comparative study Clin Exp Rheumatol 2008 26 492 497 18578976

[B7] Avčin T Cimaz R Falcini F Zulian F Martini G Simonini G Porenta-Besic V Cecchini G Borghi MO Meroni PL Prevalence and clinical significance of anti-cyclic citrullinated peptide antibodies in juvenile idiopathic arthritis Ann Rheum Dis 2002 61 608 611 10.1136/ard.61.7.608 12079901 PMC1754144

[B8] Kasapcopur O Altun S Aslan M Karaarslan S Kamburoglu-Göksel A Saribas S Arisoy N Kocazeybek B Diagnostic accuracy of anti-cyclic citrullinated peptide antibodies in juvenile idiopathic arthritis Ann Rheum Dis 2004 63 1687 1689 10.1136/ard.2003.019331 15547097 PMC1754844

[B9] Gilliam BE Reed MR Chauhan AK Dehlendorf AB Moore TL Evidence of fibrinogen as a target of citrullination in IgM rheumatoid factor-positive polyarticular juvenile idiopathic arthritis Pediatr Rheumatol Online J 2011 9 8 10.1186/1546-0096-9-8 21439056 PMC3071779

[B10] Syed RH Gilliam BE Moore TL Prevalence and significance of isotypes of anti-cyclic citrullinated peptide antibodies in juvenile idiopathic arthritis Ann Rheum Dis 2008 67 1049 1051 18556446 10.1136/ard.2007.084855

[B11] Yoshida M Tsuji M Kurosaka D Kurosaka D Yasuda J Ito Y Nishizawa T Yamada A Autoimmunity to citrullinated type II collagen in rheumatoid arthritis Mod Rheumatol 2006 16 276 281 10.1007/s10165-006-0498-y 17039307 PMC2780673

[B12] Uysal H Bockerman R Nandakumar KS Sehnert B Bajtner E Engström Å Serre G Burkhardt H Thunnissen MM Holmdahl R Structure and pathogenicity of antibodies specific for citrullinated collagen type II in experimental arthritis J Exp Med 2009 206 449 462 10.1084/jem.20081862 19204106 PMC2646582

[B13] Van Steendam K Tilleman K Deforce D The relevance of citrullinated vimentin in the production of antibodies against citrullinated proteins and the pathogenesis of rheumatoid arthritis Rheumatology 2011 50 830 837 10.1093/rheumatology/keq419 21278075 PMC3077912

[B14] Kuna AT Lamot L Miler M Harjacek M Simundic AM Vrkic N Antibodies to mutated citrullinated vimentin and antibodies to cyclic citrullinated peptides in juvenile idiopathic arthritis Clin Chem Lab Med 2009 47 1525 1530 19842993 10.1515/CCLM.2009.288

[B15] Morbach H Dannecker H Karkau T Girschick HJ Prevalence of antibodies against mutated citrullinated vimentin and cyclic citrullinated peptide in children with juvenile idiopathic arthritis Clin Exp Rheumatol 2010 28 800 20822716

[B16] Burkhardt H Sehnert B Bockerman R Engström Å Kalden JR Holmdahl R Humoral immune response to citrullinated collagen type II determinants in early rheumatoid arthritis Eur J Immunol 2005 35 1643 1652 10.1002/eji.200526000 15832289

[B17] Ioan-Facsinay A Willemze A Robinson DB Peschken CA Markland J van der Woude D Elias B Ménard HA Newkirk M Fritzler MJ Toes RE Huizinga TW El-Gabalawy HS Marked differences in fine specificity and isotype usage of the anti-citrullinated protein antibody in health and disease Arthritis Rheum 2008 58 3000 3008 10.1002/art.23763 18821680

[B18] Snir O Widhe M Hermansson M von Spee C Lindberg J Hensen S Lundberg K Engström A Venables PJ Toes RE Holmdahl R Klareskog L Malmström V Antibodies to several citrullinated antigens are enriched in the joints of rheumatoid arthritis patients Arthritis Rheum 2010 62 44 52 10.1002/art.25036 20039432

[B19] Nielen MM van der Horst AR van Schaardenburg D van der Horst-Bruinsma IE van de Stadt RJ Aarden L Dijkmans BA Hamann D Antibodies to citrullinated human fibrinogen (ACF) have diagnostic and prognostic value in early arthritis Ann Rheum Dis 2005 64 1199 1204 10.1136/ard.2004.029389 15640269 PMC1755615

[B20] Kinloch A Tatzer V Wait R Peston D Lundberg K Donatien P Moyes D Taylor PC Venables PJ Identification of citrullinated α-enolase as a candidate autoantigen in rheumatoid arthritis Arthritis Res Ther 2005 7 R1421 R1429 10.1186/ar1845 16277695 PMC1297593

[B21] Lundberg K Kinloch A Fisher BA Wegner N Wait R Charles P Mikuls TR Venables PJ Antibodies to citrullinated α-enolase peptide 1 are specific for rheumatoid arthritis and cross-react with bacterial enolase Arthritis Rheum 2008 58 3009 3019 10.1002/art.23936 18821669

[B22] Innala L Kokkonen H Eriksson C Jidell E Berglin E Rantapää-Dahlqvist S Antibodies against mutated citrullinated vimentin are a better predictor of disease activity at 24 months in early rheumatoid arthritis than antibodies against cyclic citrullinated peptides J Rheumatol 2008 35 1002 1008 18398946

[B23] van der Linden MPM van der Woude D Ioan-Facsinay A Levarht EW Stoeken-Rijsbergen G Huizinga WJ Toes RE van der Helm-van Mil AH Value of anti-modified citrullinated vimentin and third-generation anti-cyclic citrullinated peptide compared with second-generation anti-cyclic citrullinated peptide and rheumatoid factor in predicting disease outcome in undifferentiated arthritis and rheumatoid arthritis Arthritis Rheum 2009 60 2232 2241 10.1002/art.24716 19644872

[B24] Lindsley CB Janecek LL Horton WA Lindsley HB IgG anticollagen antibodies in sera of patients with childhood rheumatic diseases [abstract] Arthritis Rheum 1980 23 S56

[B25] Myers LK Higgins GC Finkel TH Reed AM Thompson JW Walton RC Hendrickson J Kerr NC Pandya-Lipman RK Shlopov BV Stastny P Postlethwaite AE Kang AH Juvenile arthritis and autoimmunity to type II collagen Arthritis Rheum 2001 44 1775 1781 10.1002/1529-0131(200108)44:8<1775::AID-ART313>3.0.CO;2-V 11508428

[B26] Barnett ML Combitchi D Trentham DE A pilot trial of oral type II collagen in the treatment of juvenile rheumatoid arthritis Arthritis Rheum 1996 39 623 628 10.1002/art.1780390413 8630112

